# Draft Genome Sequence of Neobacillus cucumis Strain T4S4, a Stevioside and Rebaudioside A Hydrolytic Strain Isolated from Tropical Forest Soil

**DOI:** 10.1128/MRA.01491-20

**Published:** 2021-05-27

**Authors:** T. Asawasriworanan, P. Harnvoravongchai, J. Somana, S. Chankhamhaengdecha, P. Ounjai

**Affiliations:** a Department of Biology, Faculty of Science, Mahidol University, Bangkok, Thailand; b Department of Biochemistry, Faculty of Science, Mahidol University, Bangkok, Thailand; Portland State University

## Abstract

Neobacillus cucumis T4S4 was isolated from montane rain forest soil in Thailand. This strain possesses the ability to hydrolyze stevioside and rebaudioside A, the two major steviol glycosides found in the stevia plant. The draft genome sequence of T4S4 yielded a circular chromosome of 5,978,437 bases with 38.9% GC content.

## ANNOUNCEMENT

A facultative anaerobic, Gram-positive, rod-shaped soil bacterium, designated strain T4S4, was isolated in Nan Province, Thailand (19°8′14″N, 100°57′25″E). T4S4 exhibits unique hydrolytic activities against stevioside and rebaudioside A (1). 16S rRNA sequence analysis of the PCR amplicon (1,416 bp) from T4S4 genomic DNA (gDNA) suggested that T4S4 belongs to the species Neobacillus cucumis, marking it as the first of the genus *Neobacillus* to harbor such activities. Here, we report the draft genome sequence of *N. cucumis* T4S4.

**FIG 1:**
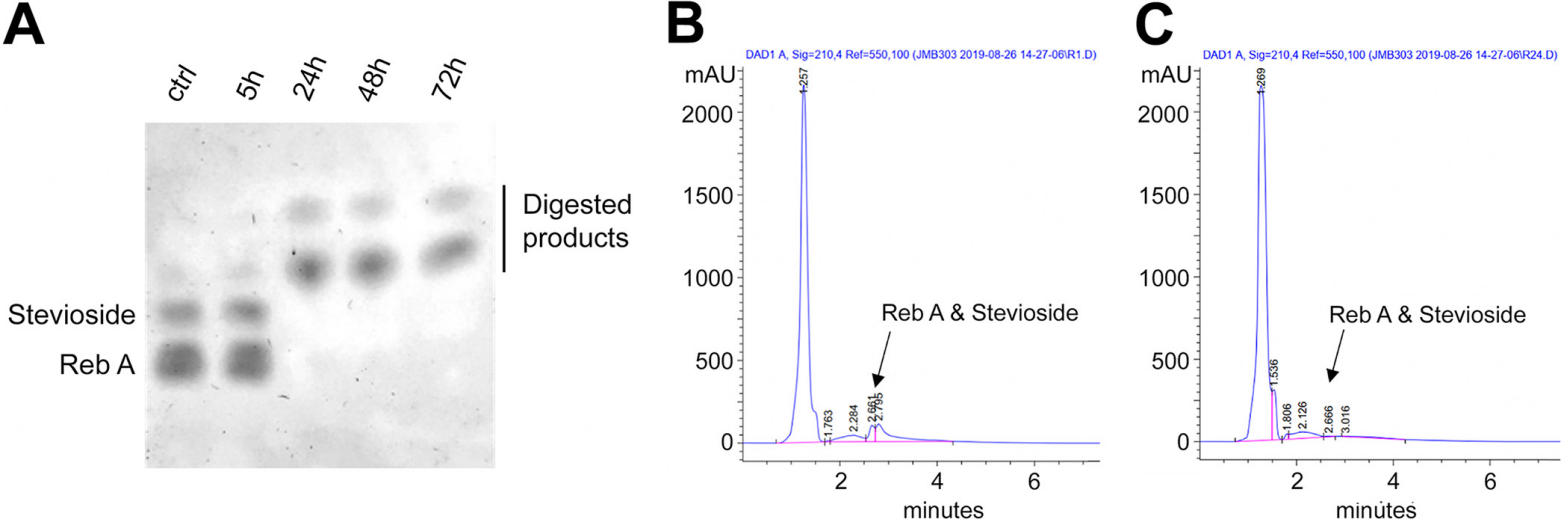
Hydrolytic activities of T4S4 against stevioside and rebaudioside A (Reb A). (A) Shifts in the stevioside and Reb A bands were observed after a 24 h cultivation. ctrl (control) = MSS. Chromatograms of stevioside and Reb A revealed hydrolytic activities by T4S4 1 h postincubation (B) and 24 h postincubation. (C) Reductions in stevioside and Reb A were observed at 24 h.

A soil specimen was sampled from at least 10 cm underneath the topsoil layer. The soil was mixed with minimal salt medium supplemented with 0.1% stevia extract (MSS) (per liter, 1 g stevia extract [Sugavia Co., Ltd., Thailand], 0.2 g yeast extract, 2 g NaNO_3_, 0.5 g MgSO_4_·7H_2_O, 0.05 g K_2_HPO_4_, 0.01 g FeSO_4_·7H_2_O, 0.02 g CaCl_2_, and 0.02 g MnSO_4_) + 25 μg/ml of amphotericin-B at a soil/medium ratio of 1:9. The mixture was microaerobically incubated at 40°C for 7 days. The culture was serially diluted and spread onto MSS plates. Hydrolytic activities against stevioside and rebaudioside A were detected using thin-layer liquid chromatography (TLC) (Fig. 1A) (2, 3). High-performance liquid chromatography (HPLC) was utilized to confirm substrate degradation (Fig. 1B and C) (2, 3).

A single colony of T4S4 was cultivated in LB broth at 37°C, with agitation at 200 rpm. Genomic DNA was extracted using the E.Z.N.A. bacterial DNA kit (Omega Bio-tek, GA) according to the manufacturer’s recommendations. The quantity and quality of the extracted DNA were evaluated using NanoDrop. Analysis of the 16S rRNA sequence (using the primers 27F and 1492R) (4) and a BLASTn search (5) identified T4S4 as *N. cucumis* with 98.66% identity. The genomic DNA was submitted for whole-genome sequencing at the Omics Center of Chulalongkorn University.

Sequencing libraries were prepared according to the Qiagen FX protocol (USA). The libraries were analyzed using the Agilent 2100 Bioanalyzer and DeNovix fluorometer before pooling at equimolar quantities. The Illumina MiSeq sequencer was used in 2 × 250-bp paired-end format, resulting in 8,645,960 raw reads (genome coverage, 250×). FastQC was used to check the raw read quality (6). Adaptors and poor-quality reads were trimmed using Trim Galore v. 0.4.4 (7). Genome assembly was conducted using SPAdes v. 3.11.1 (8). The assembled genome was annotated using Prokka v. 1.13 (9). Default parameters were used for all software.

The draft genome sequence of *N. cucumis* T4S4 consists of 52 contigs and has a total length of 5,978,437 bp, a GC content of 38.9%, and an *N*_50_ value of 358,581 bp. Genome annotation revealed a total of 5,908 genes, with 5,781 protein-coding sequences and 123 tRNAs.

### Data availability.

This whole-genome sequencing (WGS) project has been deposited at GenBank under accession number JAEHGC000000000. The version described in this paper is version JAEHGC010000000. The raw Illumina data have been submitted under SRA accession number SRR12699712 under the GenBank BioProject number PRJNA663754.

## References

[1] Ceunen S, Geuns JMC. 2013. Steviol glycosides: chemical diversity, metabolism, and function. J Nat Prod 76:1201–1228. doi:10.1021/np400203b. 23713723

[2] Udompaisarn S, Arthan D, Somana J. 2017. Development and validation of an enzymatic method to determine stevioside content from Stevia rebaudiana. J Agric Food Chem 65:3223–3229. doi:10.1021/acs.jafc.6b05793. 28343388

[3] Boonkaew B, Udompaisarn S, Arthan D, Somana J. 2019. Expression and characterization of a recombinant stevioside hydrolyzing β-glycosidase from Enterococcus casseliflavus. Protein Expr Purif 163:105449. doi:10.1016/j.pep.2019.105449. 31295559

[4] Miller CS, Handley KM, Wrighton KC, Frischkorn KR, Thomas BC, Banfield JF. 2013. Short-read assembly of full-length 16S amplicons reveals bacterial diversity in subsurface sediments. PLoS One 8:e56018. doi:10.1371/journal.pone.0056018. 23405248 PMC3566076

[5] McGinnis S, Madden TL. 2004. BLAST: at the core of a powerful and diverse set of sequence analysis tools. Nucleic Acids Res 32:W20–W25. doi:10.1093/nar/gkh435. 15215342 PMC441573

[6] Andrews S. 2010. FastQC: a quality control tool for high throughput sequence data. https://www.bioinformatics.babraham.ac.uk/projects/fastqc.

[7] Krueger F. 2015. Trim Galore: a wrapper tool around Cutadapt and FastQC to consistently apply quality and adapter trimming to FastQ files. http://www.bioinformatics.babraham.ac.uk/projects/trim_galore.

[8] Bankevich A, Nurk S, Antipov D, Gurevich AA, Dvorkin M, Kulikov AS, Lesin VM, Nikolenko SI, Pham S, Prjibelski AD, Pyshkin AV, Sirotkin AV, Vyahhi N, Tesler G, Alekseyev MA, Pevzner PA. 2012. SPAdes: a new genome assembly algorithm and its applications to single-cell sequencing. J Comput Biol 19:455–477. doi:10.1089/cmb.2012.0021. 22506599 PMC3342519

[9] Seemann T. 2014. Prokka: rapid prokaryotic genome annotation. Bioinformatics 30:2068–2069. doi:10.1093/bioinformatics/btu153. 24642063

